# P04 The evolution of antimicrobial stewardship (AMS) training for multi-professional foundation trainees (MPFT) across the East of England

**DOI:** 10.1093/jacamr/dlae217.008

**Published:** 2025-01-23

**Authors:** Naomi Fleming, Monsey McLeod, Alishah Lakha, Conor Jamieson, Gill Damant, Laura Whitney, Fiona Marshall, Sabina Khanom, Kieran Hand

**Affiliations:** NHS England, UK; NHS England, UK; NHS England, UK; NHS England, UK; NHS England, UK; NHS England, UK; NHS England, UK; NHS England, UK; NHS England, UK

## Abstract

**Background:**

MPFT include medics, pharmacists, physician associates and dental professions. Trainees frequently see cases of sepsis, urinary tract infection (UTI) and community acquired pneumonia (CAP); there are opportunities to introduce AMS learning into the optimal management of these infections. A MPFT hub event was delivered by the NHSE AMS team in 2023 in a hybrid format. Three case studies and a quality improvement (QI) presentation were delivered. Feedback was positive but suggested more interactivity e.g. a quiz would be useful and virtual participant engagement was limited.

**Methods:**

Building on this experience, two in person events in different hospitals were held in 2024 with an interactive before and after quiz. The original case studies (CS) were further developed. CS1 included sepsis, the blood culture pathway and the impact of penicillin allergy labels and was delivered by the local microbiologist, allowing local expertise and content to be added. The remaining content was delivered by the NHSE team. CS2 focused on UTI, pyelonephritis and timely IV to oral antibiotic switch and QI methodology was interwoven. CS3 discussed the management of patients with undifferentiated CAP/UTI diagnosis and *Clostridioides difficile* infection.

**Results:**

Both events were fully booked (110 registrants); 37 participants completed feedback, of which 34 were medical and 3 were pharmacists. Participants rated their overall satisfaction at 4.81/5. All participants indicated that attending the event improved their AMS knowledge. CS were scored on a scale of definitely, mostly, somewhat or not at all. 81%, 70% and 73% scored CS1, 2 and 3 definitely interesting, 92%, 84% and 84% scored CS1, 2 and 3 as definitely useful, 92%, 92% and 87% scored CS1, 2 and 3 definitely relevant to their area of work and 89%, 84% and 87% scored CS1, 2 and 3 definitely pitched correctly. Comments included ‘I loved everything from start to finish. This should be incorporated in Foundation teaching’, ‘It’s difficult to make microbiology interesting but I couldn’t fault today to be honest’ and ‘I loved how interactive the session was and how everyone was given a chance to think instead of being given the answer. I enjoyed the discussions as different trusts use different antibiotics and as some of these management options can be adopted in my current trust.’ Suggestions provided were: ‘A little bit more info on bioavailability of Abx e.g., which ones can be PO instead of IV without reducing efficacy’, ‘Would have preferred a longer session with more breaks’ and ‘Only lectures, would be nice to have other type of sessions’.

**Conclusions:**

Content was evaluated as useful, relevant and pitched correctly for medics and pharmacists, but could be more interactive. Content could be shared and used for foundation training across the UK for medical and pharmacist trainees but may need adapting for dental students. For future events planned, we will consider other pedological methods and add information on bioavailability.

